# Management strategies and survival in cutaneous B-cell lymphoma: A population based study

**DOI:** 10.1016/j.jdin.2023.06.015

**Published:** 2023-07-12

**Authors:** Kyle Lauck, Areebah S. Ahmad, Quoc-Bao D. Nguyen, Zixi Yang, Suprateek Kundu, Auris O. Huen

**Affiliations:** a Division of Dermatology, Department of Internal Medicine, Baylor University Medical Center, Dallas, Texas; b UTHealth McGovern Medical School, Houston, Texas; c Department of Dermatology, The University of Texas MD Anderson Cancer Center, Houston, Texas; d Department of Dermatology, UTHealth McGovern Medical School, Houston, Texas; e Department of Biostatistics, The University of Texas MD Anderson Cancer Center, Houston, Texas

**Keywords:** cause specific survival, chemotherapy, cutaneous B-cell lymphoma, cutaneous lymphoma, oncology, radiotherapy

*To the Editor:* Primary cutaneous B-cell lymphomas (CBCL) are a heterogenous group of extranodal non-Hodgkin lymphomas involving the skin. CBCL makes up around 20% to 25% of all cutaneous lymphomas.^1^^,^^2^ Within the spectrum of CBCL subtypes, there is a broad range of prognoses.^2^ The therapeutic management varies between watchful waiting, surgical excision, radiotherapy (RT), and/or multiagent cytotoxic chemotherapy. Evidence guiding therapy is mostly observational.^3^ Moreover, population level data are lacking, with a relative absence of data specific to CBCL therapy.^4^ The aim of this study was to examine whether there is an association at a population level between therapeutic strategy and survival when stratifying by CBCL subtype.

The Surveillance, Epidemiology, and End Results (SEER) program was explored for subtypes of CBCL with primary skin sites using corresponding International Classification of Diseases for Oncology, third edition codes. Eight subtypes were classified into indolent and aggressive categories based on their relative mortality rates. Indolent category included the following: marginal-zone B-cell lymphoma, follicular lymphoma, lymphoplasmacytic lymphoma, and small B-lymphocytic lymphoma. Aggressive category included the following: diffuse large B-cell lymphoma (DLBCL), immunoblastic DLBCL, Burkitt lymphoma, mantle cell lymphoma.

Therapeutic and survival data were extracted for patients with one of the above diagnoses. After adjusting for age, gender, and race, a multivariate cause specific survival analysis was conducted using Kaplan-Meier method with log-rank statistics and Cox proportional hazards modeling.

In total, 3188 CBCL cases were found between 1975 and 2018. Among patients with indolent CBCL, those who received chemotherapy had a significantly increased risk of death by 229% compared to those without chemotherapy (Table I, Fig 1). Among patients with aggressive CBCL subtypes, those who received RT had a significantly reduced risk of death by 56% (Table I).

**Table I:** Hazard ratios of therapeutic strategies among cutaneous B-cell lymphoma subtypes

	Cutaneous B-cell lymphoma subtype	
	Indolent	Aggressive
Therapeutic strategy		
Primary site surgery	HR = 1.20 CI (0.85-1.68) *P* = .295	HR = 0.94 CI (0.77-1.15) *P* = .523
Radiotherapy	HR = 0.74 CI (0.53-1.03) *P* = .074	HR = 0.56 CI (0.46-0.69) *P* < .01
Chemotherapy	HR = 2.29 CI (1.58-3.34) *P* < .01	HR = 1.16 CI (0.95-1.42) *P* = .134

*CI*, 95% Confidence interval; *HR*, hazard ratio.

**Fig 1:**
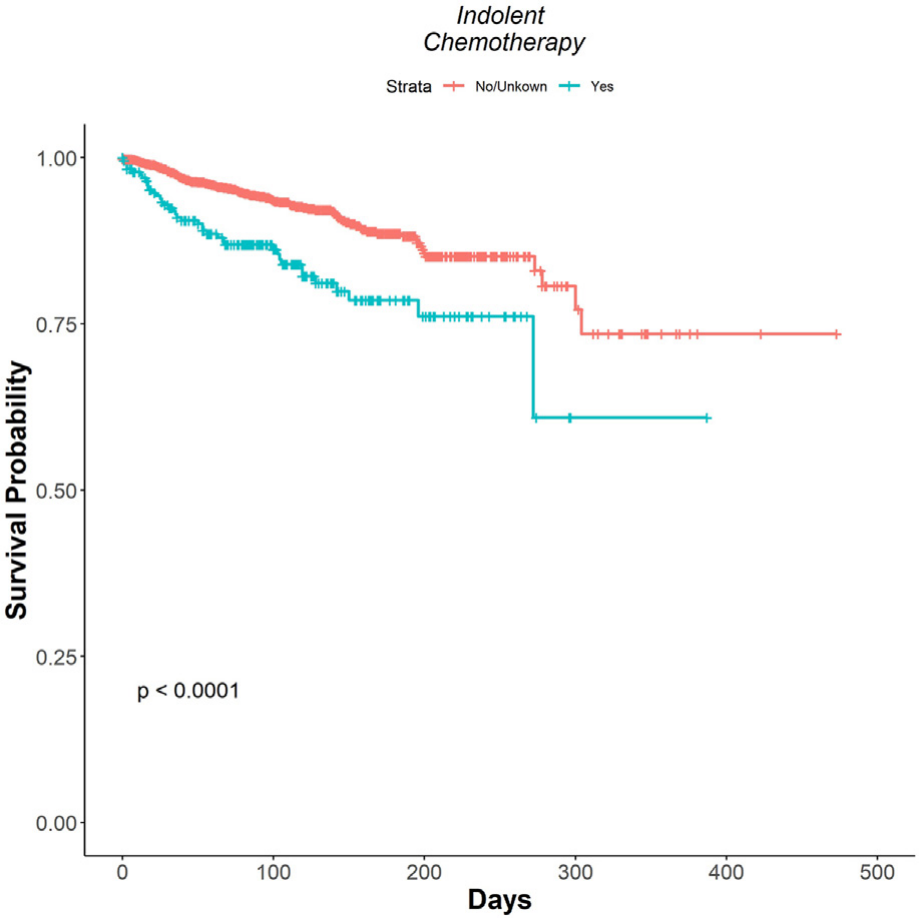
Cause specific survival analysis of patients with indolent cutaneous B-cell lymphoma subtypes receiving chemotherapy.

Based on the SEER database, patients with indolent CBCL subtypes had a higher risk of death when they received chemotherapy. Additionally, chemotherapy provided no significant benefit to survival in patients with aggressive CBCL. RT improved survival outcomes among patients with aggressive CBCL.

Limitations of SEER must be acknowledged, including the fact that some patients may exhibit systemic lymphoma with secondary cutaneous involvement. Only patients with a diagnostic code for a primary neoplasm of the skin were included, but the possibility of underdocumented systemic lymphoma exists, especially in cases seen before 1995. Another potential source of misclassification is overlap in features between different CBCL subtypes. SEER provides minimal detail on therapy timing and types of chemotherapy administered. Some individual CBCL subtypes may have different therapeutic approaches that are not comprehensively analyzed because of grouping of this population into two larger cohorts to sufficiently allow cause specific survival analysis. Due to changing classification, consistently staging patients with CBCL in SEER was not possible and may represent a confounder. However, a notable strength of this study is the large population analyzed given the rarity of this disease entity.

These results suggest uncertainty surrounding the benefit of systemic chemotherapy for patients with CBCL, especially indolent subtypes of CBCL. Skin directed therapy may be superior to systemic therapy in indolent subtypes of CBCL. In the future, confirmatory studies will assess the effects of stage of therapy and time to therapy and may add more detail to the observations reported here.

## Conflicts of interest

None disclosed.
